# Removal of power-line interference from the ECG: a review of the subtraction procedure

**DOI:** 10.1186/1475-925X-4-50

**Published:** 2005-08-23

**Authors:** Chavdar Levkov, Georgy Mihov, Ratcho Ivanov, Ivan Daskalov, Ivaylo Christov, Ivan Dotsinsky

**Affiliations:** 1 Signa Cor laboratory, Lubliana str 46, 1618 Sofia, Bulgaria; 2 Technical University of Sofia, Faculty of Electronic Engineering and Technologies (FETT), Kliment Ohridski str. 8, 1000 Sofia, Bulgaria; 3 Centre of Biomedical Engineering, Bulgarian Academy of Sciences, Acad. G. Bonchev str., blok 105, 1113 Sofia, Bulgaria

## Abstract

**Background:**

Modern biomedical amplifiers have a very high common mode rejection ratio. Nevertheless, recordings are often contaminated by residual power-line interference. Traditional analogue and digital filters are known to suppress ECG components near to the power-line frequency. Different types of digital notch filters are widely used despite their inherent contradiction: tolerable signal distortion needs a narrow frequency band, which leads to ineffective filtering in cases of larger frequency deviation of the interference. Adaptive filtering introduces unacceptable transient response time, especially after steep and large QRS complexes. Other available techniques such as Fourier transform do not work in real time. The subtraction procedure is found to cope better with this problem.

**Method:**

The subtraction procedure was developed some two decades ago, and almost totally eliminates power-line interference from the ECG signal. This procedure does not affect the signal frequency components around the interfering frequency. Digital filtering is applied on linear segments of the signal to remove the interference components. These interference components are stored and further subtracted from the signal wherever non-linear segments are encountered.

**Results:**

Modifications of the subtraction procedure have been used in thousands of ECG instruments and computer-aided systems. Other work has extended this procedure to almost all possible cases of sampling rate and interference frequency variation. Improved structure of the on-line procedure has worked successfully regardless of the multiplicity between the sampling rate and the interference frequency. Such flexibility is due to the use of specific filter modules.

**Conclusion:**

The subtraction procedure has largely proved advantageous over other methods for power-line interference cancellation in ECG signals.

## Background

Modern biomedical amplifiers have very high common mode rejection ratio (CMRR), with commercial ECG instruments manifesting values up to 120 dB. Nevertheless, recordings are often contaminated by residual power-line (PL) interference. This is due to differences in the electrode impedances and to stray currents through the patient and the cables. Thus, the common mode voltage is transformed into a false differential signal [1-4] that cannot be suppressed even by an infinitely high CMRR. The problems become more complicated if the instrument has a floating input to increase patient safety [5,6].

CMRR of a commercial ECG instrument is typically measured under laboratory conditions using generators with low impedance output and short connecting wires. Thus, a claim of CMRR > 60 ÷ 70 dB in the real world of ECG acquisition is without legitimate basis.

Any residual PL interference may interfere with the correct delineation of ECG wave boundaries [7] and corrupt the proper function of automatic ECG analysis. The interference can also disturb the correct measurement of RR intervals, which is the basis for heart rate variability analysis.

Hardware solutions have been developed to increase the actual CMRR by equalization of the cable shield and the right leg potentials [4]. This reduces the influence of stray currents through the body, but the efficiency obtained is not sufficient to significantly reduce the interference.

Traditional analogue and digital filters are known to suppress ECG components near the PL frequency. Different types of digital notch filters are widely used [8,9] despite their inherent contradiction: tolerable signal distortion needs a narrow frequency band, which leads to ineffective filtering with larger PL frequency deviation. Moreover, the resulting transient time is often unacceptably long. Hamilton [10] compares the convergence times of adaptive and non-adaptive notch filters. Both introduce significant distortion in the QRS and ST-segment portions due to the filter ringing. Soo-Chang and Chien-Cheng [8] try to reduce to some extent the transient response time by using vector projection to find better initial values for IIR notch filters. Yoo et al. [11] propose a hardware notch filter with adaptive central frequency to follow the PL frequency changes, thus defining a narrower bandwidth. Filters with various Q factors have been tried. However, the resulting signal distortion cannot be correctly assessed because of the reduced scale of the examples provided [11].

Instead, the condition was simulated in the MATLAB environment [12]. A synthesized ECG signal (without noise) was mixed with constant 1 mV_p-p _50 Hz interference and processed by notch filters with bandwidths: 49–51, 49.5–50.5, and 49.9–50.1 Hz. This 1 Hz bandwidth is one order of magnitude narrower than that used by Yoo et al. [11]. Acceptable distortion was found only with the 49.9 ÷ 50.1 Hz filter, but after an exclusively long tail of about 12 s. This adaptation period reappeared with abrupt power-line frequency change of 0.2 Hz, despite a synchronised identical shift of the filter centre frequency.

Ringing is also present when spectral components of the interference are removed from the ECG signals using the Fourier transform [13]. Furthermore, this transform does not work in real time.

Mitov [14] uses parabolic detrending of ECG to estimate the signal components with frequencies corresponding to PL interference by using the discrete Fourier transform, to approximate averaged interference values, which are subtracted from the contaminated signal. No results with frequency-modulated interference are presented in the publication.

The interference may be suppressed by adaptive filtering [15-17]. However, this technique introduces unacceptable transient response time, especially following signals of steep and high amplitude, e.g. the QRS complex.

Kumaravel and Nithiyanandam [18] reported interference cancellation by an off-line working genetic algorithm.

Some authors do not present the results of their algorithms correctly or clearly enough to use for interference removal. Sometimes the original signal is not presented [19], no differences between original and processed signals are shown [20], and the performance is measured by the error square instead of amplitude differences [21].

## Method

The subtraction procedure for PL elimination was first elaborated some two decades ago [22]. This procedure does not affect the ECG components neighbouring the PL frequency. This theoretical study is carried out for the basic PL frequency, but the conclusions are also valid for its harmonics and, consequently, for an arbitrary interference waveform. The efficiency of the procedure does not depend on the amplitude of the interference, as long as the amplifier is not saturated. Moreover, the procedure copes successfully with changes in amplitude and frequency of the interference. The procedure has been continuously improved over the years [12,13,23-32], and implemented in thousands ECG instruments and computer-aided systems [33,34]. Similar approaches have also been published by other researchers [35-41].

### Principles

The subtraction procedure is applied originally with sampling frequency *f_S_*, a multiple of, and hardware synchronized with the PL frequency *f_PL_*. The procedure consists of the following steps [22]:

• ECG segments with frequency band near zero are continuously detected using an appropriate criterion. They are referred to as linear segments and are found mainly in the PQ and TP intervals, but also in sufficiently long straight parts of the R and T waves.

• The samples of these segments are moving averaged, i.e., subjected to a linear phase comb filter [42] with first zero set at *f_PL_*. Thus, the filtered samples do not contain interference.

• Interference amplitudes, called corrections, are calculated for each of the phase-locked samples, *n*, in the PL period, *T_PL_*, by subtracting the filtered samples from the corresponding ones of the contaminated (original) ECG signal.

• The set of corrections obtained is continually updated in linear segments and used in non-linear segments (usually around QRS complexes and high-amplitude T waves) to subtract the interference from the original ECG signal.

**Figure 1:**
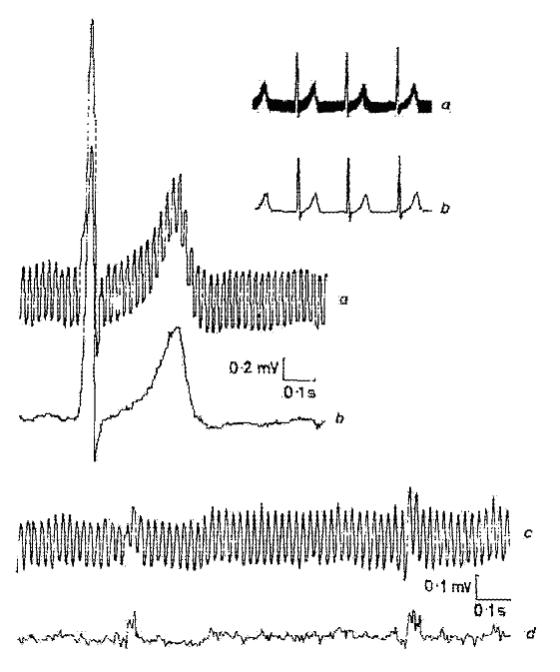
One of the first results obtained by the subtraction procedure.

One of the first results obtained by the subtraction method is shown in Fig. 1 [22]. Interference was added to a clean simulated ECG signal in order to evaluate the errors and the efficiency of the method.

### Linear criterion

A linear criterion, *Cr*, usually corresponds to the second difference of the signal (mathematical evaluation of the linearity). The first *Cr *[22] is defined in the following manner. Six consecutive first differences, *FD_i_*, are calculated using signal samples, *X_i_*, spaced at one *T_PL_*:

*FD_i _*= *X_i+n _*- *X_i_*, *for i = 1 ... 6 *    (1)

The PL interference in the first differences is suppressed if *n *= *f_S_/f_PL_*. In this case *n *= 5, since the procedure was developed initially for rated *f_PL _*= 50 Hz and *f_S _*= 250 Hz. Furthermore, the maximum *FD*_*max *_and minimum *FD*_*min *_values are taken to determine *Cr*:

*Cr *= | *FD*_*max *_- *FD*_*min *_| <*M*,     (2)

where *M *is the threshold value.

**Figure 2:**
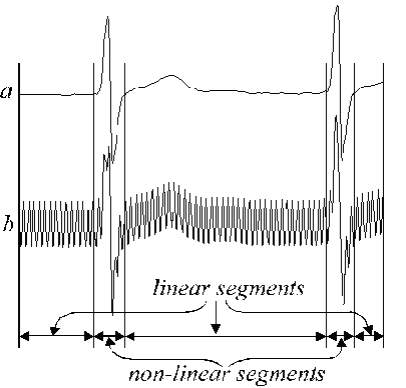
Typical linear and non-linear segments in real ECG signal.

**Figure 3:**
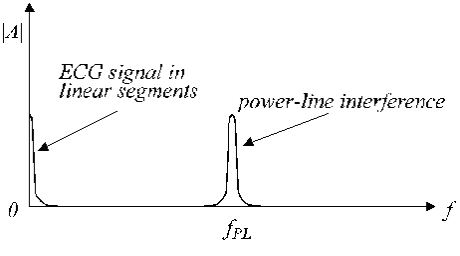
Approximate frequency spectrum of a linear segment.

Typical linear and non-linear segments are shown in Fig. 2. Real ECG signal (trace *a*) is superimposed by interference (trace *b*). The linear segments include low frequency signal and power-line frequency components. An approximate frequency spectrum of such linear segments is shown in Fig. 3.

This criterion works accurately, but can hardly be applied in real time because its relatively slow implementation. This drawback is overcome by Christov and Dotsinsky [23] who use a modified criterion of just two subsequent differences.

*Cr *= | *FD*_*i*+1 _- *FD*_*i *_| <*M*.     (3)

The first sample, which does not fulfill equation (3), is associated with the beginning of a non-liner segment. In the non-linear to linear transition, equation (3) should be satisfied consecutively *n *times in order to avoid premature detection of the linear segment. The criterion is implemented in real time for *f*_*S *_= 400 Hz and *n *= 8.

Later, Dotsinsky and Daskalov [13] defined the criterion as two non-subsequent differences:

*Cr *= |*FD*_*i*+*k *_- *FD*_*i *_| <*M*, *for k *>*1 *    (4)

This approach makes the transition from linear to non-linear segment more precise.

### Compensation of PL amplitude variations

The more frequently the corrections are updated, the better compensation of the amplitude variations of the PL is achieved. Therefore, the linear criterion threshold, M, has to be reasonably less restrictive so that the errors, committed by averaging some segments that depart from the ideal linear signal, are smaller than the errors, that will appear if M initiates sporadic updating of the correction. Initially, M was fixed at 160 μV [22]. Later, heuristic values were found to be M = 150 μV [23] and M = 100 μV [13].

### Linear filtering

For odd sample number *n *= 2*m *+ 1 in one period of the PL interference, the filtered value:



is phase-coincident with the non-filtered one.

In case of even number *n *= 2*m*, the two values are phase-shifted by a half of the sample period:



but become in-phase coincidence using the formula



It is possible to take for averaging every second, third or *q*^*th *^sample if *n/q *is integer. Depending on whether *n/q *is odd or even, equation (7) or (8) is used, respectively.





A special case of maximum sample reducing arises with *q *= *n*/2 [28]. The corresponding formula:



is called 'three-points' filter. In addition to equation (8), the following formula



can also be applied if *q *is even. In case of *q *= *n*/2, the filter becomes 'two-points' and is represented by:



Reduced sample number in a period of the interference will lead to enhanced steep slope of the comb filter lobes and will shorten the computation time. However, these 'advantages' must be assessed carefully in order not to violate the Nyquist rule with a large amount of the third harmonic present. The other harmonics are not taken into consideration since the highest odd harmonics are usually suppressed by low-pass filters with cut-off in the range of 100–150 Hz, while the even ones are practically absent because of the precise pole manufacturing of the electric power station generators.

### Compensation of PL frequency variation

The allowed deviation from the rated PL frequency is limited in some countries up to 1% by the standards. In practice, deviation is oftentimes higher. Kumaravel *et al*. [43] reported for variation of 3%. McManus *et al. *[44] found considerable changes in the interference frequency, which is superimposed on recordings taken from the Common Standards for Electrocardiography (CSE) database.

Frequency variations lead to a special case of non-multiple sampling with real *n*, instead of integer one. This complication can be bypassed if the deviations are detected by continuous hardware measurement of *f*_*PL *_and corrected by small adjustments of the sample interval *t*_*S *_around its rated (*R*) value, *t*_*RS *_= *T*_*RPL*_/*n *(here, *T*_*RPL *_= 20 ms is the rated *T*_*PL *_for *f*_*RPL *_= 50 Hz). For *f*_*PL*_, deviation between 49.5 and 50.1 Hz, the *t*_*S *_variations are in the range of 1%, and consequently they do not introduce errors beyond the accepted measuring accuracy of parameters that are usually used for automatic ECG classification.

A first approach associates the triggering of each first sample, *S*_*1*_, of the sequences *S*_*k *_(*k *= 1, 2...*n*) in the periods *T*_*PL *_with arbitrary chosen but constant amplitude of the PL voltage. The next samples, *S*_*k *_(*k *= 2...*n*), are spaced at *t*_*S*_, which is obtained by *t*_*S *_= *T*_*RTL*_/*n*. For 50 Hz, and *n *= 5, *t*_*S *_= 4 ms. Two types of errors committed using this approach are studied by Dotsinsky and Daskalov [13]. The first, due to inter-sample irregularities, may reach 1% at *f*_*S *_= 400 Hz and 1.2% at *f*_*S *_= 250 Hz, in case of 1% deviation around the *f*_*RPL*_. The second type of error does not exceed 3% and is a consequence of the additionally shifted location of the filtered sample.

Dotsinsky and Daskalov [13] reported an improved approach. The ongoing period *T*_*PL *_is measured and divided by *n*. The obtained *t*_*S *_is used in the subsequent *T*_*PL*_.

### Efficiency assessment of the procedure

**Figure 4:**
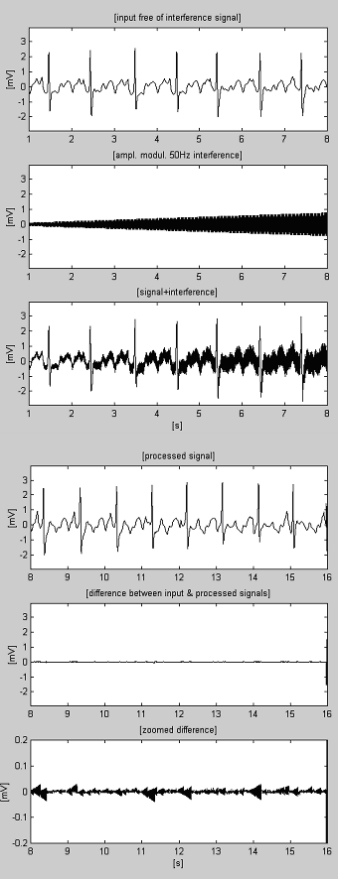
Processed AHA 3004d1 recording.

**Figure 5:**
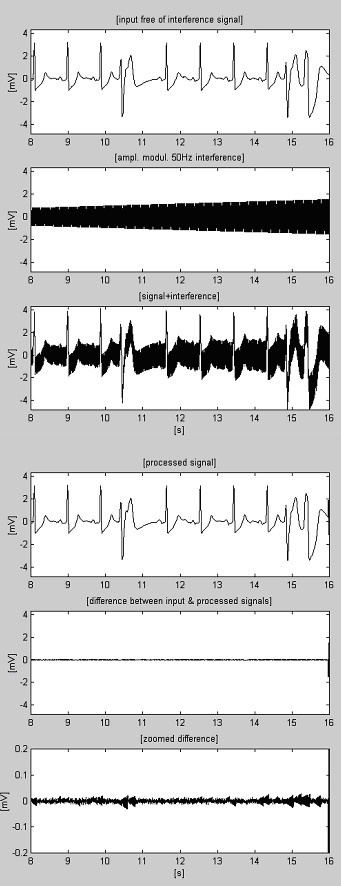
Processed AHA 6007d1 recording.

Subtraction procedure examples are shown in Fig. 4 and Fig. 5. The ECG signals are taken from the American Heart Association database. The signals are notch filtered to suppress the 60 Hz (PL frequency in the US) interference. Then, the signals are mixed with 50 Hz interference, amplitude modulated from 0 through 3.2 mV_p-p _by a slew rate of 200 μVs^-1^. The traces are identified as follows: i) input signal; ii) synthesized interference; iii) mixed signal; iv) processed signal; v) difference between original and processed signals and vi) zoomed difference. Actually, the discarded components also include electromyographic (EMG) and other noises. A non-suppressed part of the signal, together with small residual interference and distortions due to compromise with the *M *value are also present in the differences.

**Figure 6:**
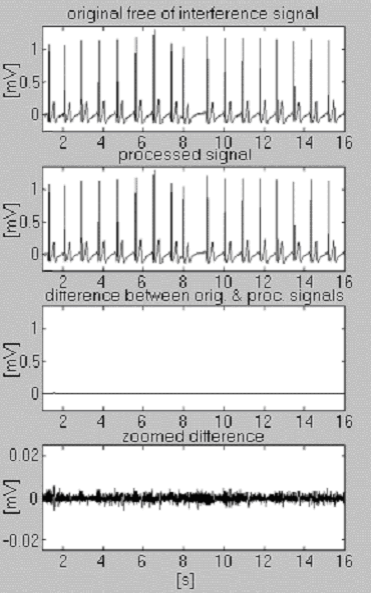
Processing of conditionally 'clean' signal.

**Figure 7:**
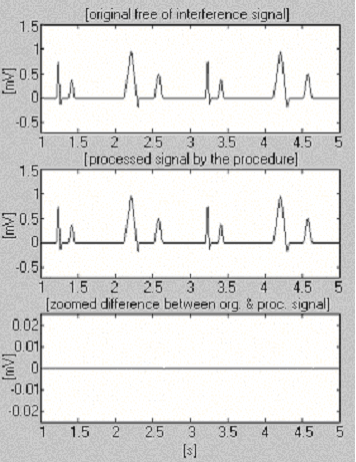
Processing of synthesized signal.

**Figure 8:**
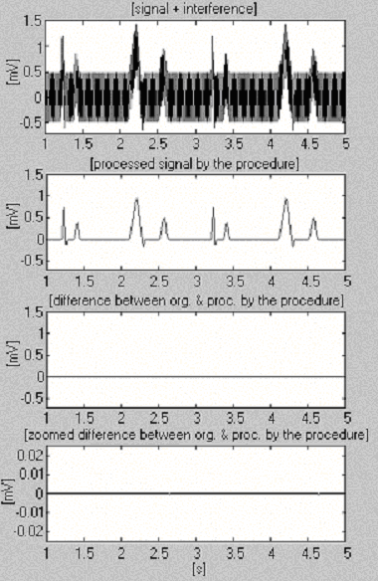
Processed synthesized signal+interference.

Two signals are used to assess the efficiency of the subtraction procedure with respect to the interference only. The first, taken from our own database, is called conditionally 'clean' (Fig. 6). The result shows small differences between input and processed signals, visually due to the noise presented in the input. This result is verified with the second synthesized signal, which does not contain any disturbances (Fig. 7). As can be seen, no distortions are introduced. The same synthesized signal is superimposed by interference and processed (Fig. 8). No residual interference can be found.

### Influence of EMG noise

**Figure 9:**
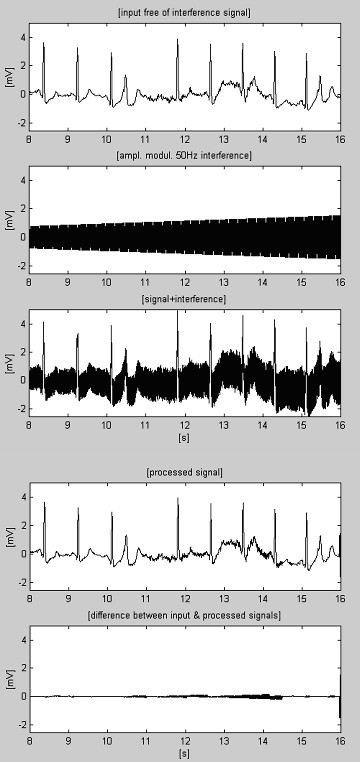
Processed ECG signal+EMG noise+interference.

Sometimes EMG noise is so high that the linear segment finding is hampered. As a consequence, inaccurate corrections, which do not correspond to the last change of the interference amplitude, will cause errors (see the residual noise between the 11^th ^and 14^th ^s in Fig. 9).

**Figure 10:**
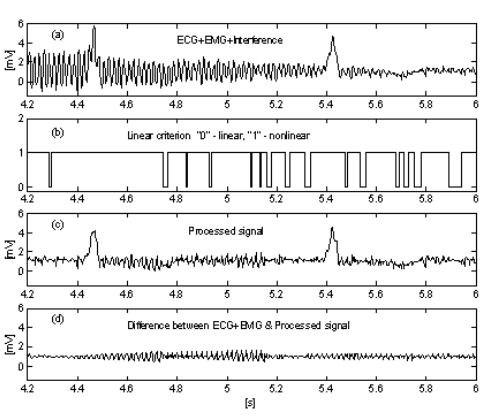
Subtraction of interference+EMG noise without parallel buffer.

**Figure 11:**
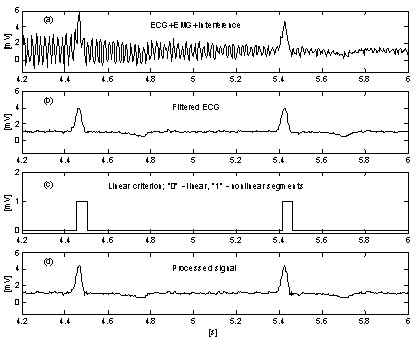
Subtraction of interference+EMG noise with parallel buffer.

A very simple approach for suppression of EMG noise influence on the procedure introduces an additional parallel buffer where ongoing portions of the signal are unconditionally averaged [31]. This buffer is used for accurate linearity detection. Fig. 10 and Fig. 11 show the comparison of results without and with the parallel buffer. The traces '*a*' represent ECG signal mixed with interference and EMG noise. The traces '*b*' in Fig. 10 and '*c*' in Fig. 11 indicate transitions from linear to non-linear segments (on-off functions). As can be seen, the averaged signal part in Fig. 10 is very limited. As a consequence, the procedure efficiency is reduced (Fig. 10c and 10d. In contrast, the parallel buffer allows detection of long linear segments (Fig. 11c and the residual noise in the processed signal (Fig. 11d is low. However, the noise is not totally suppressed because a part of it participates in the correction calculation.

**Figure 12:**
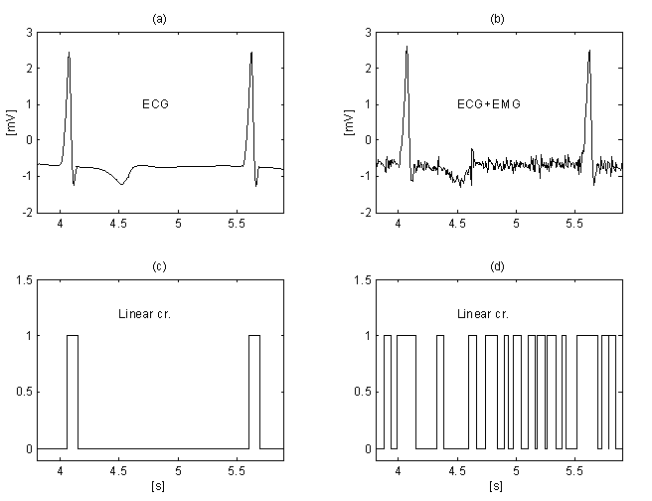
Noise levels S_NL _(traces c and d) in 'clean' ECG signal (trace a) and EMG contaminated signal (trace b), respectively.

**Figure 13:**
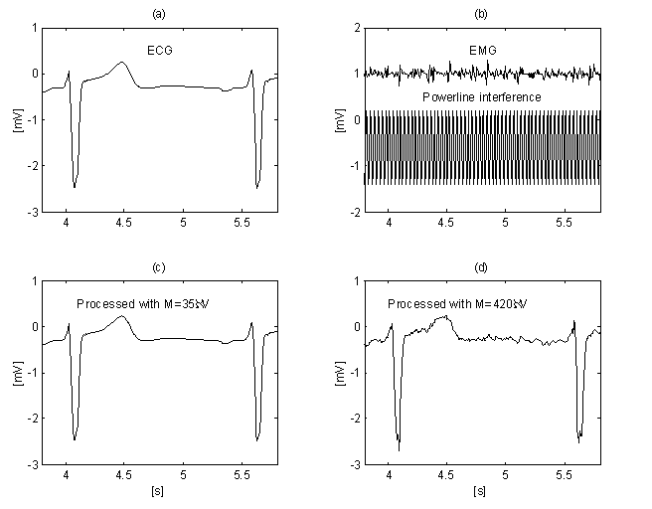
Interference+EMG noise suppression using adaptive threshold M: 'clean signal' (a) processed with M = 35 μV (c) and contaminated signal (b) subjected to the procedure with M = 420 μV (d).

Further improvement in EMG noise suppression is obtained by Christov [29] by using adaptive threshold value *M*, which is calculated with respect to the noise/signal ratio *Rt*, defined as *Rt *= *S*_*NL*_/*S*_*F*_, where the noise level *S*_*NL *_equals the summary width of the non-linear segments in an epoch *S*_*F*_, approximately corresponding to the RR interval under consideration. Linearity search with a criterion of *M *= 150 μV for 'clean' ECG (Fig. 12a) and for the same signal, but contaminated with EMG noise (Fig. 12b) show different *S*_*NL*_, (Fig. 12c and 12d). The method is developed in MATLAB environment. The initial threshold *M *is chosen equal to 30 μV. Then, it is gradually increased until *Rt *reaches 10%, after which the subtraction procedure is started. The level *Rt *≤ 0.1 value is suggested by the ratio 'QRS width versus its RR interval', which is usually around 10%. The elimination of both interference and EMG noise can be observed in Fig. 13b and 13d, where *M *= 420 μV is used. For comparison, the 'clean' ECG signal is processed with *M *= 35 μV (Fig. 13a and 13c).

Linear segments cannot be regularly found in patients with atrial and ventricular fibrillation. However, the total preservation of the wave shape is not necessary for fibrillation detection and therefore, all kinds of traditional filters may be applied.

### Interference suppression in high-resolution ECG

The subtraction procedure is not directly applicable to the body-surface His-ECG, as the low amplitude and relatively low frequency His-wave can not be distinguished in linear segments. Thus, the His-wave will be, in practice, suppressed or even removed from the signal. The EMG noise is usually of higher amplitude and with much higher frequency content compared to the surface His-wave. Therefore, simple change of the threshold value, *M*, does not result in acceptable delineation of linear and nonlinear segments.

**Figure 14:**
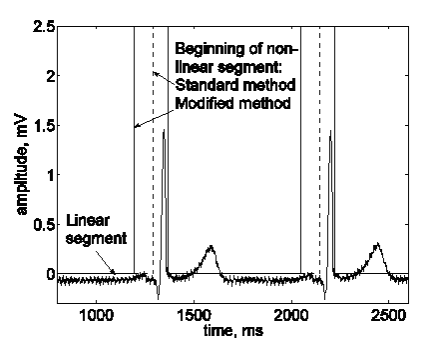
The beginning of a non-linear segment including the QRS complex was shifted by default 100 ms to the left, to include the zone where the His-bundle wave is expected to appear.

Bazhina *et al*. [45,46] implemented the following modification. The beginning of the detected non-linear segment before a QRS complex is shifted 100 ms to the left, thus defining the His-wave region as a non-linear segment by default (Fig. 14).

The subtraction procedure and three other methods: notch filters, spectral interpolation [47], and regression subtraction [35] are tested against minimal distortion of the original signal [45]. The subtraction and the regression-subtraction procedures proved to be the best, as Baratta *et al*. [35] use a similar concept for noise estimation in linear segments. Regression-subtraction deals poorly with amplitude changes of the interference within the current segment.

### Case of battery-supplied devices and computer aided ECG systems

The hardware measurement of *f*_*PL*_, necessary for compensation of the interference frequency modulation, is not feasible in battery-supplied devices and in some computer aided ECG systems. Dotsinsky and Stoyanov [12] studied the range of frequency changes of interference with constant amplitude, for which the residual part is restricted to acceptable levels without use of synchronized sampling. They found that residual interference below 20 μV_p-p _could be obtained with the procedure by: i) interference amplitude ≤ 0.4 mV_p-p _and ii) frequency change with a rate ≤ 0.0125 Hzs^-1^. Since such requirements for the power-supply can often be exceeded, a software interference measuring was developed.

**Figure 15:**
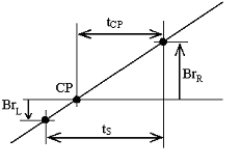
Interference zero crossing.



The ECG signal is processed initially by a 49–51 Hz band-pass filter. The amplitudes of two adjacent samples, *Br*_*L *_and *Br*_*R*_, taken from a positive-going slope of the interference, located below and above the zero line, are measured (Fig. 15). The distance, *t*_*CP*_, between the crossing point *CP *and the right sample, *Br*_*R*_, is computed continually by:

In case of *T*_*PL *_change, *t*_*S *_is redefined using



**Figure 16:**
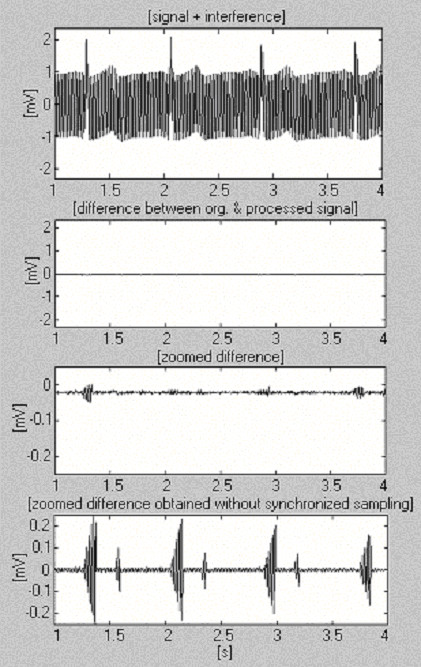
Subtraction procedure using software power-line frequency measurement.

This approach was implemented in the MATLAB environment. For sampling frequency *f*_*S *_= 500 Hz and *f*_*RPL *_= 50 Hz, *n *is equal to 10. The product *kn *determines the time recommended to elapse before calculating and substituting new *t*_*CP*,*i *+ *kn *_for the previous *t*_*CP*,*i*_. Fig. 16 shows a processed 1 mV ECG signal after being mixed by interference with 2 mV_p-p _constant amplitude and extremely fast varying by 1 Hz per 8 s frequency (first trace). To assess the efficiency obtained, the zoomed difference without synchronized sampling (last trace) is also presented.

The next logical step to be taken consists of: i) keeping the rated *t*_*S *_of the ECG instrument, ii) re-sampling the signal according to the ongoing measured *f*_*PL *_in order to eliminate the interference and iii) returning to the rated *t*_*S*_. The first results of such an approach are highly promising [48]. Thus, the software compensation of the variable *f*_*PL*_, as well as a total implementation of the subtraction procedure in an instrument, including automatic adjustment for *f*_*RPL *_of 50 or 60 Hz, will be completed regardless of the hardware circuits and the corresponding software.

### Automatic adaptation to the rated PL frequency

A common program for alternative interference subtraction in 50 and 60 Hz environment leads to non-multiple sampling, i.e. to real *n*. Widely used values of *t*_*S *_for *f*_*RPL *_= 50 Hz, such as 250, 500 and 1000 Hz, correspond to irrational *n *of 4.1(6), 8.3(3) and 4.1(6) if 60 Hz interference has to be eliminated. In the inverse case, *f*_*S *_= 360 Hz requires *n *= 7.2. Rounded values n* are unacceptable to use, since they would introduce considerable error.

A very simple solution not needing *f*_*S *_change was found by Dotsinsky and Stoyanov [30]. The original procedure applies a comb filter over one period, *T*_*PL*_, of the interference. Thus, the program runs faster. Generally, *n *may be taken from *k *> 1 entire periods. The procedure is operated if:

*n *= *kT*_*PL*_/*t*_*S *_is an integer.

**Figure 17:**
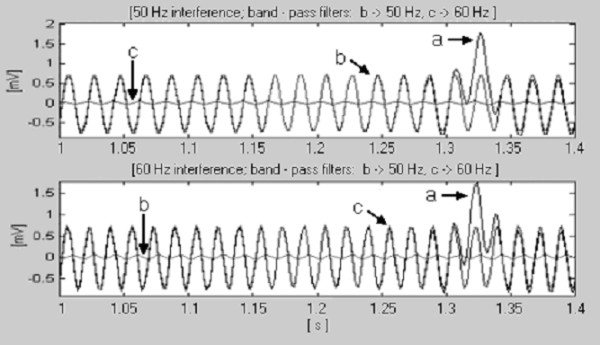
Detection of the rated power-line frequency, a) part of QRS complex, b) 50 Hz interference and c) 60 Hz interference.

For *t*_*S *_= 2 ms (*f*_*S *_= 500 Hz) and *f*_*RPL *_= 50 Hz, the smallest value of *k *satisfying equation (14) is really *k *= 1. However, in case of 60 Hz, *k *equals 3. Zeros associated with the sub-harmonics 20 and 40 Hz will appear too but they have no influence on the procedure. Therefore, it is quite enough to switch *n *between 10 (*k *= 1) and 25 (*k *= 3) in order to operate with both interferences. For this purpose two digital band-pass filters check the incoming signal. Fig. 17 shows that the filter with frequency band overlapping the interference generates an order of magnitude higher output signal than the other filter.

### Theoretical procedure development

The theory of the subtraction procedure was developed further by Mihov [27], Levkov and Mihov [28], and Mihov *et al*. [32]. They proposed four types of filters, implemented in a generalized structure that may overcome the problems with almost all cases of non-multiple sampling, including interference frequency variations, without using synchronized AD conversion.

The so-called D-filter in multiple sampling is defined as is *Cr *in equation (2), where the second difference, *D*_*i*_, is obtained with *FD*s that are spaced at one *T*_*PL*_:

*D*_*i *_= (*X*_*i+n *_- *X*_*i*_) - (*X*_*i *_- *X*_*i-n*_) = *X*_*i-n *_- 2*X*_*i *_+ *X*_*i+n *_    (15)

**Figure 18:**
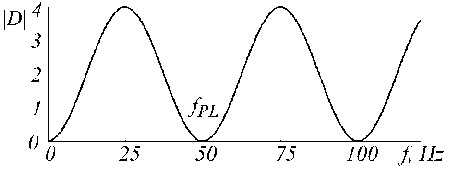
Transfer function of D-filter for f_PL _= 50 Hz.

The transfer function of the D-filter has zeros at *f *= 0 and *f *= *f*_*PL *_Hz, which is shown equal to 50 Hz in Fig. 18.

**Figure 19:**
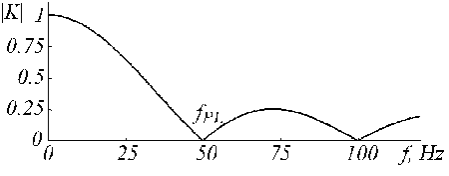
Transfer function of K-filter for f_PL _= 50 Hz and n = 5.

The K-filter describes the moving average presented by equations (5) and (6). Its transfer function is given in Fig. 19 for *n *= 5 in case of odd multiplicity.

The equation used for ongoing calculation of the interference components:

*B*_*i *_= *X*_*i *_- *Y*_*i *_    (16)

actually defines a digital filter called (1-K)-filter.

Furthermore, the filters are re-defined for non-multiple sampling, and *f*_*S *_= 250 Hz in conjunction with f_*RPL *_= 60 Hz is taken in consideration to illustrate the software improvement.

In order to preserve the transfer function zeros, the D-filter has to be subtracted with a correction filter with zero at *f *= 0 and gain of *D*_*RPL *_at *f *= *f*_*RPL*_, equal to the gain of the D-filter for the same frequency, *f*_*RPL*_. The correction filter synthesis is based on a three-points auxiliary filter given by the equation:



where (*n*/2)* is the rounded value of *n*/2.

**Figure 20:**
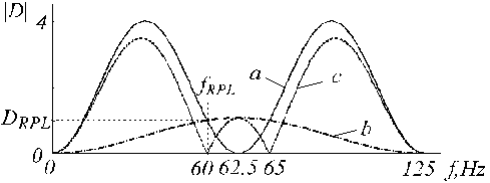
Transfer functions of a) D-filter, b) auxiliary correction filter and c) corrected D-filter.

Since *A*_*RPL *_is the gain of the auxiliary filter for *f *= *f*_*RPL*_, the correction filter is multiplied by the ratio *D*_*RPL*_/*A*_*RPL*_. Using the corresponding transfer functions, *D*_*RPL *_and *A*_*RPL *_are computed in advance by:



Finally, the corrected D*-filter is presented as



and is shown in Fig. 20 by trace '*c*', where traces '*a*' and '*b*' are the D-filter and the correction filter, respectively.

The transfer function of the K-filter must preserve zero for *f *= *f*_*RPL*_, unity gain for *f *= 0 and linear phase response. The procedure of the K-filter correction is similar to the previous one. An auxiliary filter is given by the formula used for corrections computation:

*A*_*i *_= *X*_*i *_- *Y*_*i*_,     (20)

The filter gain is equal to 1 - *K*_*RPL *_for *f *= *f*_*RPL*_, where *K*_*RPL *_is the K-filter gain for the same frequency *f*_*RPL*_. The auxiliary filter is multiplied by *K*_*RPL*_/(1 - *K*_*RPL*_) and subtracted from the K-filter. The equation for the corrected K*-filter is:



The constant *K*_*RPL *_can be estimated by:



for odd or even multiplicity, respectively.

**Figure 21:**
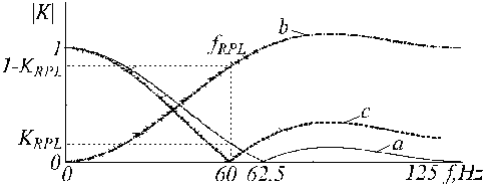
Transfer function of a) K-filter, b) auxiliary correction filter and c) corrected K-filter.

An example of K-filter correction is shown in Fig. 21, where traces '*a*', '*b*' and '*c*' represent the primary K-filter, the auxiliary filter, and the corrected K*-filter.

**Figure 22:**
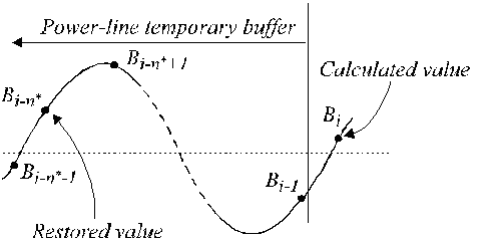
Restored values in the temporary buffer.



In case of non-multiple sampling, a phase difference appears between the ongoing ECG samples and the interference components *B*_*i *_(equation 16) usually located in a temporary first-in-first-out (FIFO) buffer. Therefore, *B*_*i *_must be modified being subtracted from the ECG samples during non-linear segments. The compensation procedure is relatively complicated. Fig. 22 shows the contents of the temporary buffer. The current interference sample, B_i-n_*, does not coincide with the restored sample, *B*_*i*_. Its amplitude must be recalculated in order to compensate the phase difference between them. This is accomplished by a new filter with linear phase response and unity gain for *f *= *f*_*RPL*_, denoted as the B-filter. It is synthesized from the known K-filter, with a window equal to the interference period. In case of odd n*, it can be described as:

where *K*_*RPL *_is the gain for the interference of the averaging filter given by equation (22).

**Figure 23:**
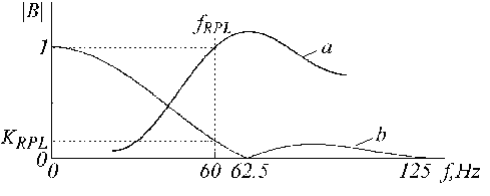
Transfer functions of a) B-filter and b) the known K-filter.

The restored buffer value *B*_*i *_can be calculated by:



In case of even n*:



The B-filter transfer function is shown in Fig. 23.

**Figure 24:**
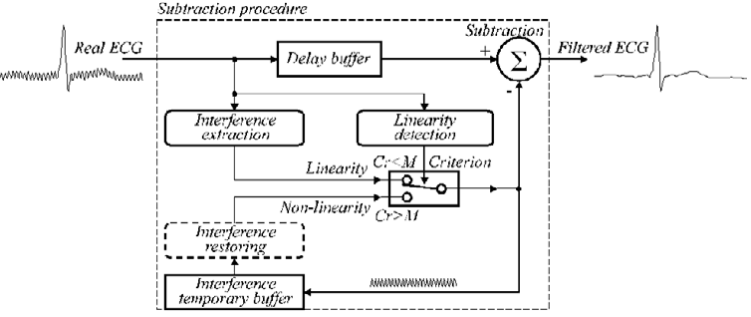
Generalized structure of the subtraction procedure.

The generalized structure is presented in Fig. 24, where the modules of the subtraction procedure are as follows:

• *Linearity detection*. D-filter is applied to evaluate the linearity of each signal sample neighbourhood.

• *Interference extraction*. (1-K)-filter is used to calculate the interference component.

• *Criterion*. The condition *Cr *<*M *sends either extracted or restored PL interference to *Subtraction*.

• *Interference temporary buffer*. The extracted or restored interference component used as correction in non-linear segment is saved at the position locked with the ongoing phase of the power-line interference.

• *Interference restoring*. B-filter is called in case of non-multiple sampling in order to restore the true correction values, which have to be subtracted from the input signal samples in non-linear segments.

• *Delay buffer*. Compensates the delay, which appears with the D-filter and (1-K)-filter and is imperative if the procedure is run in quasi-real time. Otherwise, the buffer could be disregarded.

• *Subtraction*. Extracted or restored interference value is subtracted from the delayed input signal to output 'clean' ECG signal. In case of non-linearity both *Interference extraction *and *Subtraction *implement the K-filter.

**Figure 25:**
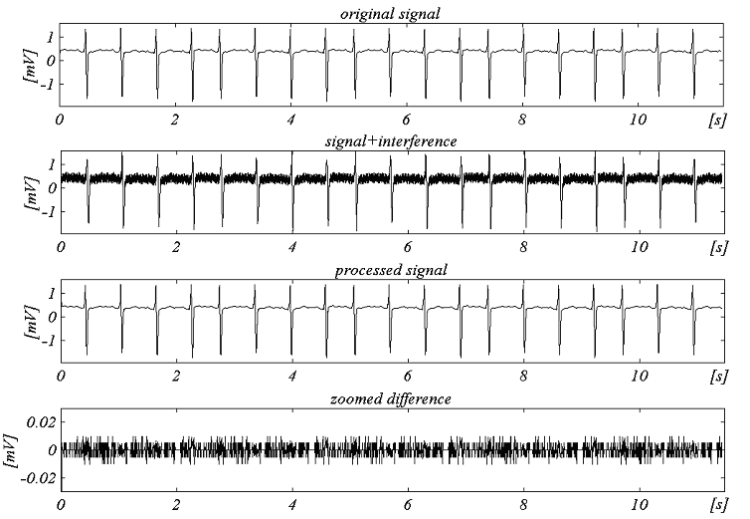
Example for non-multiple sampling with f_RPL _= 60 Hz and f_S _= 250 Hz.

An improved algorithm according to the generalized structure has been tested off-line. The results for *f*_*S *_= 250 Hz and *f*_*RPL *_= 60 Hz are shown in Fig. 25.

## Conclusion

As first elaborated two decades ago and continually improved since then, the subtraction procedure eliminates power-line interference from the ECG signal without affecting its spectrum. The procedure operates successfully even with amplitude and frequency deviations of the interference. The frequency deviations are first compensated by hardware measurement of the power-line frequency. Software measurement of the interference period was developed for battery supplied units and some ECG modules connected to personal computers.

The improved structure of on-line going subtraction procedure leads to its extended implementation regardless of multiplicity between sampling rate and interference frequency. The structure flexibility is due to the introduced filtering modules, which are called into use depending on the type of sampling.

The presented analysis of the subtraction procedure and the different types of notch filters confirms the advantages of this method for interference cancellation in ECG signals.
